# 

**Published:** 1895-03-30

**Authors:** 


**Notices of Births, Deaths, and Marriages** are inserted in this column at a charge of 2s. 6d. for an announcement not exceeding four lines.

**Notice to Advertisers and Subscribers.**

All Advertisements, Orders for Copies of The Hospital and Business Communications should be addressed to **The Manager, NOT TO THE EDITOR,** The Hospital, 428, Strand, London, W.C.

The Cost of Subscription, post free, is as follows:—Per week, 2½d.; per quarter, 2s. 8d.; per half-year, 5s. 3d.; per annum, 10s. 6d. Foreign:—Per Annum, 15s. 2d.; payable, in all cases, in advance.

**NOTICE TO CORRESPONDENTS.**

All MS., Letters, Books for Review, and other matters intended for the Editor should be addressed The Editor, The Lodge, Porchester Square, London, W.

The Editor cannot undertake to return rejected MS., even when accompanied by stamped directed envelope.

**"Burdett's Hospital Annual," 1895,**

Ready Immediately.

Sixth year of publication. About 1000 pp. Scarlet cloth, gilt lettered. Price 5s. A most complete guide to British, Colonial, Indian, and American Hospitals, Dispensaries, Nursing and Convalescent Institutions, and Asylums. A few copies of the "Annual" for 1894 may still be obtained on application to the Publishers, The Scientific Press (Limited), 428, Strand, London, W.C.

**NOTICE.—The Index for Vol. XVII (ending March 30th, 1895) will be on sale at the Office of this Journal in a few days, Price 2½d., post free.**

### SCRAPS AND GLEANINGS.

The Queen has sent a present of old linen to the Royal Free Hospital.

A deficit of £1,000 exists on the accounts of the South Devon and East Cornwall Hospital.

A fund is being started, we learn, in New York for the manufacture and sale of diphtheria antitoxin.

The Queen has sent a present of 100 lbs. of cast linen for the use of the patients in the London Hospital.

The Irish Distressed Ladies' Fund has received a donation of £52 10s. from the Worshipful Company of Salters.

The Duke and Duchess of York have been pleased to accord their patronage to the Hospital for Consumption, Brompton.

Messrs. Nobles and Hoare have contributed £250 towards the special appeal fund of St. Thomas's Hospital for the opening of closed wards.

A consumptive home, the second of its kind in America, is being built at Verbank, New York. This is proposed to accommodate 300 patients.

A new Medical Association has been formed in Rome. The committee is a very influential one, and the association has for its object the upholding of the honour of the profession and the guardianship of its interests.

The medical students of the Glasgow University have presented Professor John Yule Mackay, M.D., of University College, Dundee, with a gold watch in recognition of their appreciation of his services as Demonstrator and Lecturer.

Dr. Kennedy Dalziel has been appointed to occupy the chair of surgery in Anderson's College, Glasgow, in place of Dr. James Dunlop. He holds several important surgical appointments, and has an excellent reputation as a lecturer.

A cheque for £500 has been received from Mr. George Palmer to wipe off the debt existing on the funds of the Royal Berkshire Hospital. A further sum of £600 will still be required if the construction of the operating theatre be finally decided upon.

Great improvements have been effected at the Silloth (Carlisle Convalescent Institution during the past year, the men's portion being considerably altered and extended. The institution will very shortly have accommodation for 15 more patients.

A grand bazaar will be inaugurated in May in the City of London on behalf of the British and Foreign Sailors' Society, Princess Louise having consented to open the same. Miss Böcker, the only lady saved from the "Elbe," will assist at the German stall.

Two concerts of an exceptionally novel and attractive character were given recently at the Assembly Rooms, Cork, for the benefit of the County and City of Cork Hospital for Women and Children, on which institution there exists at present a debt of £400.

A convalescent home is much needed at Portsmouth, and it is hoped that operations will shortly be commenced to construct one. Sir John Baker has given £500. Towards the same charitable purpose a lady, who wishes to remain anonymous, has contributed £1,500.

Patients from twenty parishes have been received in the Charnwood Forest Convalescent Home. The new premises have been found most convenient, and well adapted for their purpose, the augmentation in the number of beds permitting patients to be admitted at once.

The committee of the North Durham Eye Infirmary in presenting their 59th report do so with the gratifying sense that the labours of many years have been crowned with such success that it is possible to report the new building site and furnishing as paid for, and the institution as entirely free from debt.

At the Oswestry and Ellesmere Cottage Hospital the nurses' quarters are being enlarged. Mr. Humphreys, of Hanley Hall, has contributed £500 towards this object, and legacies of £200 and £300 have been received respectively from the late Mrs. Parry, of Barmouth, and Miss Griffiths, of Llanymynech.

A large convalescent home, the gift of Mr. Henry Harben, J.P., is being commenced at Littlehampton. It has been designed by Mr. Frederick Wheeler, of 22, Chancery Lane, and will be fitted with every modern convenience, including lighting by electricity. Most of the bedrooms are to be for one bed only.

In the last annual report of the Pendlebury Children's Hospital at Manchester, reference is made with great satisfaction to the generous responses of the public to a special appeal, and to the endeavour, by the newly-instituted ladies' committee, to wipe off the heavy debt, and to almost cover the year's expenditure.

A new cot has been established in the National Children's Hospital, Dublin, the "Beauchamp Cot," supported by Mrs. Langley West. The committee wish the public to know that for the annual donation, subscription or collection of £12, such cot will be named after the donor, and cease to be an expense to the institution.

At the last meeting of the managers of the Edinburgh Royal Infirmary reference was made to the number of patients applying for relief at the institution who had no claim on its charitable support. Well-to-do people should remember, in return for the relief thus afforded them gratuitously, that they should contribute according to their means to the funds of the infirmary.

### BOOKS RECEIVED.

THACKER, SPINCK, AND CO., CALCUTTA.
"Anglo-Urdu Medical Handbook." George Small.
SWAN SONNENSCHEIN.
"Physiological Psychology." Dr. Theodore Ziehen.
JOHN WRIGHT AND CO., BRISTOL.
"Medical Annual."

**Periodicals and Pamphlets.**—Leeds Hospital Magazine, Toilers of the Deep, Studies from the Yale Psychological Laboratory, Edinburgh Medical Record, The Bookseller, New York Medical Record.

### THE STUDENT OF MEDICINE.

### APPOINTMENTS.

A. E. Aust-Lawrence, M.D., reappointed Physician-Accoucheur to the Bristol General Hospital for a third period of ten years. R. W. Brew, B.A.Dub., M.B., B.Ch., appointed Medical Officer for the Enniskerry Dispensary District. W. King Bullmore, M.D., appointed Medical Officer to the Workhouse of the Falmouth Union. G. N. Coombes, L.R.C.P., L.R.C.S.Edin., L.F.P.S.Glasg., appointed Medical Officer to the Government of His Highness the Rajah of Cochin, Southern India. Alice M. Corthorn, M.B.Lond., appointed Assistant Medical Officer at the North-Western Fever Hospital, Haverstock Hill, N.W. W. P. Counsellor, M.D.Durh., L.R.C.P.I., reappointed Medical Officer of Health to the Clitheroe Rural District Council. E. A. Dent, M.B., C.M.Edin., appointed House Surgeon to the Devonshire Hospital, Buxton. H. W. Dun, M.B., C.M.Edin., appointed Junior Resident Assistant Medical Officer and Dispenser to the Sheffield Union Workhouse. J. C. Edgar, M.R.C.S., L.R.C.P., appointed Resident Clinical Assistant to the Barnes Convalescent Hospital, Cheadle, near Manchester. F. Edmunds, M.R.C.S., L.R.C.P.Lond., appointed Medical Officer and Public Vaccinator to the Chesterfield Workhouse and District. Eleonora L. Fleury, M.D.R.U.I., appointed Assistant Medical Officer to the Richmond Lunatic Asylum, Dublin. F. I. Flower, M.R.C.S.Eng., reappointed Medical Officer of Health to the Warminster District Council. F. D. Grayson, M.R.C.S.Eng., reappointed Medical Officer for the Hadleigh and Rayleigh District of the Rochford Union. Dr. Haworth, appointed Police Surgeon at Darwen. W. W. Hoare, M.R.C.S.Eng., L.R.C.P.Lond., appointed Chief Medical Officer to the Hospitals and Estates of the New London Borneo Tobacco Company, British North Borneo. F. W. Lindsay, L.R.C.P., L.R.C.S.Edin., reappointed Honorary Surgeon to the Victoria Eye and Ear Hospital, Hereford. Dr. Messiter, appointed Medical Officer of Health to the Isle of Axholme Rural District Council. W. Nettle, M.R.C.S.Eng., L.S.A., reappointed Medical Officer of Health to the Liskeard Town Council. T. W. Nunn, F.R.C.S.Eng., Consulting Surgeon to the Middlesex Hospital, appointed Consulting Surgeon to the London Skin Hospital, Fitzroy Square, W. Dr. J. Patterson,

### THE STUDENT OF MEDICINE—*continued.*

appointed Medical Officer for the Berriew, Llanmerewig, Llandyssil, and Forden Districts of the Forden Union. H. Pulford, M.A., M.B., C.M.Cantab., appointed Third Assistant Medical Officer to the Worcester County and City Lunatic Asylum. D. V. Rees, L.R.C.P.Lond., M.R.C.S., appointed Medical Officer of Health for the Brecon Urban District. C. S. Rothsay, M.R.C.S., L.S.A., appointed Medical Superintendent to the Leicestershire and Rutland Asylum. W. C. Sharpe, M.B.Edin., appointed Senior Physician to Smedley's Hydropathic Institution, Matlock. E. M. Skerritt, M.D.Lond., F.R.C.P., reappointed Physician to the Bristol General Hospital for a third period of ten years. R. Thompson, M.D., B.S., &c., appointed Honorary Ophthalmic Surgeon to the Ipswich Hospital, Queensland, and Visiting Ophthalmic Surgeon to the Blind Institute, Brisbane, Queensland. D. G. P. Thomson, M.B., C.M.Edin., appointed Medical Officer of Health to the Penrith District Council. F. H. Westmacott, F.R.C.S.Eng., reappointed Resident Medical Officer to the Barnes Convalescent Hospital, Cheadle, near Manchester. A. Whytt, M.B., C.M.Edin., appointed Assistant House Surgeon to the Devonshire Hospital, Buxton.

### VACANCIES.

The following vacancies are announced this week, the amount of salary in each case being given after the name of the institution:

#### Resident Medical Officers.

Bethlem Hospital.—Two Resident Clinical Assistants; doubly qualified. Appointment for six months. Will be provided with such apartments, rations, washing, and attendance as the Committee shall consider reasonable. Applications, endorsed "Clinical Assistantship," to the Treasurer, at Bethlem Hospital, before March 31st.

East London Hospital for Children, Glamis Road, Shadwell, E.—House Physician. Board, lodging, &c., provided; no salary. Applications to the Secretary by April 8th.

General Hospital, Birmingham.—Two Assistant House Surgeons. Must possess surgical qualifications and be registered. Appointment for six months. No salary, but residence, board, and washing provided. Applications and testimonials, with certificate of registration, to Howard J. Collins, House Governor, by March 30th.

Leeds Union.—Assistant Medical Officer for the Workhouse Schools and Infirmary in Beckett Street, Leeds. Doubly qualified, unmarried, and not above 35 years of age. Applications (on forms to be obtained of the Clerk), endorsed "Assistant Medical Officer," to Jno. King, Clerk, Union Offices, East Parade, Leeds, by April 1st.

Leicestershire and Rutland Asylum.—Assistant Medical Officer. Unmarried, and between the ages of 25 and 30. Salary £150, increasing £10 yearly to £200, with board, lodging, and washing. Applications to W. J. Freer, Clerk to the Visitors, 10, New Street, Leicester, by April 3rd.

Lincoln County Hospital.—House Surgeon. Under 30 years of age, and unmarried. Salary £100 per annum, with board, lodging, and washing. Applications to the Secretary by April 6th.

Miller Hospital and Royal Kent Dispensary, Greenwich Road, S.E.—Junior Resident Medical Officer. Salary £30 per annum, with board, residence, and washing. Post tenable in the first instance for six months. Applications to the Honorary Secretary by April 4th.

Surrey County Lunatic Asylum, Brookwood, near Woking, Surrey.—Second Assistant Medical Officer. Salary £120 per annum, with board, lodging, attendance, laundry, but no beer. Applications to the Medical Superintendent by April 1st.

Tancred's Charities.—Two Studentships in Divinity at Christ College, and one Studentship in Physic at Gonville and Caius College, Cambridge. Each student will receive £50 a year and a share in the surplus rents and profits (the stipend not to exceed £100 a year); the student in Physic until admission to the degree of Bachelor of Medicine, and for three years afterwards, or for such shorter terms as may be determined by authority. Candidates must be natives of Great Britain, members of the Church of England, unmarried, and not below the age of 16 or above the age of 22. Forms of petition and all information may be obtained from Mr. George Edgar Frére, 28, Lincoln's Inn Fields, W.C., Clerk to the Tancred's Charities, to whom petitions must be sent by April 20th.

Taunton and Somerset Hospital.—House Surgeon; doubly qualified. Appointment for three years. Salary £100 per annum, with board, lodging, and washing. Applications to J. H. Biddulph Pinchard, Secretary, 13, Hammet Street, Taunton, by April 4th.

#### Non-Resident Medical Officers.

Bloomsbury Dispensary, 12, Bloomsbury Street, W.C.—Physician. Must be F. or M.R.C.P.Lond, Honorarium £105 per annum. Applications to the Secretary by April 2nd.

Brighton Throat and Ear Hospital, 28, Queen's Road, Brighton.—Non-Resident House Surgeon. Salary at the rate of £52 per annum. Applications to the Secretary by April 3rd.

London School of Medicine for Women.—Demonstrator of Physiology. Applications to the Secretary, 30, Handel Street, Brunswick Square, W.C., by March 31st.

North-Eastern Hospital for Children, Hackney Road, Shoreditch, N.E.—Physician. Must be F. or M.R.C.P.Lond. Applications to T. Glenton-Kerr, Secretary, at the Office, 27, Clement's Lane, E.C., by April 16th.

Royal College of Physicians.—Milroy Lecturer. Applications to the Registrar, Royal College of Physicians, Pall Mall East, S.W., by April 9th.

### UNIVERSITY OF DUBLIN.

At the Spring Commencements in Hilary Term, held (according to custom) on Shrove Tuesday, February 26th, in the Theatre of Trinity College, the following licences and degrees in Medicine were conferred by the University Caput in the presence of the Senate:

Licentiati in Medicina, in Chirurgia, et in Arte Obstetricia.—W. R. Davy, A. H Middleton.

Baccalaureus in Medicina.—J. R. Mallins.

Baccalaurei in Medicina, in Chirurgia, et in Arte Obstetricia.—W. Ayres, A. A. Cooper, F. R. Cosgrave, H. A. Engelbach, H. F. S. Langstaff, C. J. Martin, Rev. H. C. Packard.

Doctores in Medicina.—H. Croly, A. T. Geoghegan, W. Hamilton, R. M. Hewitt, C. B. Scott, A. E. Taylor.

### Medical and Administrative Appointments.

### NORTH-EASTERN HOSPITAL for CHILDREN,
HACKNEY ROAD, SHOREDITCH, N.E.

T. GLENTON-KERR, Secretary.

Office, 27, Clement's Lane, E.C.,

